# Vitamin D in Corona Virus Disease 2019 (COVID-19) Related Multisystem Inflammatory Syndrome in Children (MIS-C)

**DOI:** 10.3389/fimmu.2021.648546

**Published:** 2021-03-08

**Authors:** Gavriela Feketea, Vasiliki Vlacha, Ioana Corina Bocsan, Emilia Vassilopoulou, Luminita Aurelia Stanciu, Mihnea Zdrenghea

**Affiliations:** ^1^Department of Hematology, “Iuliu Hatieganu” University of Medicine and Pharmacy, Cluj-Napoca, Romania; ^2^Department of Pediatrics, Pediatric Allergy Outpatient Clinic, “Karamandaneio”, Children Hospital, Patras, Greece; ^3^Department of Early Years Learning and Care, University of Ioannina, Ioannina, Greece; ^4^Department of Pharmacology, Toxicology and Clinical Pharmacology, “Iuliu Hatieganu” University of Medicine and Pharmacy, Cluj-Napoca, Romania; ^5^Department of Nutritional Sciences and Dietetics International Hellenic University, Thessaloniki, Greece; ^6^National Heart and Lung Institute, Imperial College London, London, United Kingdom; ^7^Ion Chiricuta Oncology Institute, Cluj-Napoca, Romania

**Keywords:** vitamin D, 25(OH)D3, SARS-CoV-2 infection, COVID-19, multisystem, Kawasaki syndrome-like

## Abstract

Multisystem Inflammatory Syndrome in children (MIS-C) is a rare but devastating complication of coronavirus disease 19 (COVID-19). The development of prognostic biomarkers and more importantly the implementation of new treatment modalities would have a significant impact in clinical practice regarding the outcome of MIS-C. Vitamin D could be a potential candidate. In this mini review we analyze the immunomodulatory role of vitamin D in viral infections and specifically in COVID-19. We also examine the current literature regarding the association of vitamin D with MIS-C and Kawasaki disease. The vitamin D was evaluated not only as a biomarker but also as a nutritional supplement. We concluded that vitamin D levels could be valuable in predicting severe forms of MIS-C and correction of abnormal levels in severe MIS-C may influences its evolution. 25-hydroxyvitamin D3 [25(OH)D3] supplementation raising serum [25(OH)D] concentrations potentially have a favorable effect in reducing the severity of MIS-C in certain circumstances. Further studies are needed to confirm these results.

## Key Points—Questions

Is vitamin D level a potential biomarker associated with prevalence and severity of Multisystem Inflammatory Syndrome in children (MIS-C)?

Could vitamin D supplementation affect severity and/or clinical course of MIS-C?

## Meaning

The Multisystemic Inflammatory Syndrome in Children (MIS-C) is a devastating complication of COVID-19. Vitamin D as an easily measurable compound and also readily available and without significant side effects when it is administered could have a significant impact not only as a biomarker of the disease progression but also as a supplement for favorable evolution of patients with MIS-C.

## Introduction

**Coronavirus disease 19** (COVID-19) morbidity and severity in children as well as the incidence of Multisystem Inflammatory Syndrome in Children (MIS-C) clearly vary around the world. A number of factors may account for those differences. Several studies have reported a variety of comorbidities including overweight, asthma and ethnic origin black or Asian, and minority ethnic (BAME) groups as risk factors for MIS-C (1). These conditions are independently associated with Vitamin D (vit D) deficiency and that could be a possible explication for higher MIS-C incidence in these children. Vit D can reduce the risk of infections, through several mechanisms: (i) inducing cathelicidins and defensins that can lower viral replication rates, (ii). reducing concentrations of pro-inflammatory cytokines and (iii) increasing concentrations of anti-inflammatory cytokines (2). A negative correlation has been observed between mean level of vit D and the number of COVID-19 cases/1 M, in each country (3).

Identification of the children who are at risk to develop MIS-C after an asymptomatic/ mild COVID-19 or the ones exposed to severe acute respirator syndrome coronavirus 2 (SARS-CoV-2), and those at risk for a severe MIS-C, could help pediatricians to improve their medical management.

Observational studies have resulted in a proposed link between reduced levels of the circulating form of vit D, 25-hydroxyvitamin D [25(OH)D], and severe or critical infection of SARS-CoV-2 in adults (4).

Additionally, there is evidence of association between low levels of vit D and Kawasaki diseases in children (5). Kawasaki disease is a syndrome resulted from an excessive immune response to an infection. During the COVID-19 pandemic several cases of children with Kawasaki-like syndrome or MIS-C have been identified (6–8).

The objective of this mini-review was to evaluate the evidence regarding the association of vit D status and MIS-C.

### Literature Review Strategy and Methods

A literature search has been performed in PubMed, Embase, Web of science and MedRxiv for relevant publications about the vit D and Coronavirus infection and Kawasaki disease published up to 30^th^ December 2020. Keyword used were vit D in combination with coronavirus infection, SARS-CoV-2 infection, COVID-19, MIS-C, multisystem inflammatory syndrome, Kawasaki disease, Kawasaki-like syndrome. Information was derived from selected reviews and original articles published in peer-reviewed journals, from preliminary reports of works that have not yet been certified by peer review. Also, reference tracking was carried out to identify other relevant articles, which were not found during the initial searching. Additionally, current guidelines of International and National Health Organizations were retrieved and summarized. Almost all the published studies until now, regarding relationship between COVID-19 and vit D status included adult patients, and data referring to children and adolescents are extremely limited. Due to a reduce number of publications referring to vit D status in children infected by SARS-CoV-2, the authors also collected information from adults. The search using the words MIS-C and vitamin D revealed no studies in PubMed, Web of science, Embase and MedRxiv. Using the other keywords, the initial search yielded 73 articles, of which, after elimination of duplicates and screening of their titles and abstracts, 7 studies were considered relevant to this review.

### Multisystem Inflammatory Syndrome in Children

Despite of low susceptibility to SARS-CoV-2 infection, especially those younger than 14 years, children are still at risk of infection (9).The prevalence of SARS-CoV-2 in children who are asymptomatic varied from 0 to 2.2% but there is a strong association between this prevalence and contemporaneous weekly incidence of COVID-19 in the general population (10). The children with SARS-CoV-2 infection usually are asymptomatic or suffer mild or moderate illness. It has been reported that the prevalence of severe or critical disease is 10.6, 7.3% and about 4% in children aged <1 year, 1–5years and 6–15 year, respectively (11). Few months after the onset of the pandemic, a series of reports around the world described clusters of children and adolescents presenting with a life-threatening, hyperinflammatory syndrome, named Kawasaki-like syndrome (8, 12), Pediatric inflammatory multisystem syndrome temporally associated with SARS-CoV-2 (PIMS-TS) (13) or Multisystem Inflammatory Syndrome in Children (MIS-C) (14). The pathogenesis of MIS-C is still unknown, although it has been suggested that this syndrome occurs while the immune system is activated against the SARS-CoV-2 virus (15). The host immune system in pediatric patients responses excessively to SARS-CoV-2 infections and lead to the multisystem inflammatory syndrome in children (MIS-C). MIS-C is a new childhood disease linked to SARS-CoV-2 infection. It is a dangerous systemic inflammation characterized by fever, abdominal symptoms, conjunctivitis, and rash which appear three to 4 weeks after the initial infection (16). The laboratory evidence of inflammation has been defined by one or more of the following biomarkers: an elevated C-reactive protein (CRP), erythrocyte sedimentation rate (ESR), fibrinogen, procalcitonin, d-dimer, ferritin, lactic acid dehydrogenase (LDH), or interleukin 6 (IL-6), elevated neutrophils, reduced lymphocytes and low albumin (17). Testing for SARS-CoV-2 infection including RT-PCR, antigen test and IgM/IgG antibody is suggested when COVID-19 or MIS-C is suspected (9).

A recent published study demonstrated that MIS-C hyperinflammation differs from severe acute COVID-19 hyperinflammation of adults, as well from the original Kawasaki disease (18). Consiglio et al. found that elevated IL-6, IL-17A, CXCL10 contributed the most to the cytokine storm of MIS-C, while IL-17A an important cytokine in Kawasaki disease, was significantly lower in MIS-C (18).

A systematic review compared characteristics between MIS-C and pediatric confirmed COVID-19 cases, found that the level of inflammation experienced in MIS-C outweigh the inflammation related to COVID-19 (16).

### Vitamin D: Vitamin—Hormone. Similarity With Other Steroid Hormones

Despite that its name is “vitamin,” in fact vit D is a fat-soluble steroid pre-hormone produced in skin from UV-B exposure (19). Hormones are defined as chemical signals synthesized and secreted into the bloodstream. They act on distant tissues usually in a regulatory fashion. Their action is often associated with binding soluble proteins (20). Steroid hormones are the sex steroids (estrogens, progesterone -female and androgens -male), the mineralo-corticoids, the glucocorticoids, and the vit D with its daughter metabolites (21).

Vit D3 (cholecalciferol) is synthetized in the skin under UV-B radiation from the sun but also derived from intake of animal food and vit D3 supplements, while vit D2 (ergochalciferol) is derived only from intake of vegetable food and vit D2 supplementation. Both are initially hydroxylated in the liver by (CYP2R1, CYP27A1, CYP3A4), resulting the inactive circulating form 25-hydroxyvitamin D [25(OH)D, calcidiol]. This form is then hydroxylated in the kidney by the 1α-hydroxylase enzyme [1α(OH)ase, CYP27B1] into calcitriol, the active form of vit D (22). The immune cells are able to locally convert 25(OH)D3 into its active form−1,25(OH)2D3 (19). 1,25(OH)2D3 (1,25-dihydroxyvitamin D3, calcitriol) acts as a circulating hormone and is the most potent natural ligand of the vit D receptor (VDR) of target cells. Also, 1,25(OH)2D3 has been described as an immunomodulator targeting various immune cells, including monocytes, macrophages, dendritic cells (DCs), as well as T-lymphocytes and B-lymphocytes. For that reason, 1,25(OH)2D3 has characterized as modulator of both innate and adaptive immune responses (23). Severe infections, including COVID-19, can lead to consumption of vit D intracellularly during the immune processes. As a result of that the utilization is faster than the production.

### Vitamin D and Infections in Children

#### Relationship Between Vitamin D and Viral Infections in Children

Kassas et al. shown that lower concentration of serum vit D may be significantly associated with neonatal pneumonia. The authors concluded that the vit D level could predict the need for mechanical ventilation and the duration of hospitalization in neonates with pneumonia (24). In a randomized clinical trial (RCT) conducted among children aged 1 through 5 years, the participants were randomized to receive 2,000 IU/d of vitamin D oral supplementation (high-dose group) or 400 IU/d (standard-dose group) for a minimum of 4 months during the winter and has been found no statistically significant difference in several laboratory-confirmed upper respiratory tract infections between the two groups (25). Recently, an updated meta-analysis of data from RCTs of vit D for the prevention of respiratory infections in children and adults reported an overall protective effect of the intervention with modest statistically significance and found that the daily administration of standard doses for up to 12 months was the most beneficial regime (26). While previous meta-analysis shown a protective effect of vit D supplementation among those with the lowest baseline vit D level, this was not observed in this update (27). Forno et al. performed a randomized, double-blind, placebo-controlled clinical trial of vit D_3_ supplementation in high-risk children with asthma aged 6 to 16 years taking low-dose inhaled corticosteroids. The subjects had serum 25(OH)D levels <30 ng/ml. Compared to placebo, vit D_3_ supplementation did not significantly improve the time to a first viral-induced severe exacerbation (28).

#### Relationship Between Vitamin D and SARS-CoV-2 Infection in Children

Since the beginning of the pandemic, despite the lack of direct evidence of an effect of vit D on SARS-CoV-2 infections, it had been suggested the vit D supplementation could be a safe, inexpensive and readily available modifiable intervention for COVID-19 (29). Subsequently, a growing evidence has been published regarding adults, suggesting that serum vit D levels could be valuable in preventing SARS-CoV-2 infection and predicting not only COVID-19 mortality rates but also linearly predicting COVID-19 illness severity (30). However, according to others, there is not enough evidence on the association between vit D levels and COVID-19 severity and mortality (31).

An association but not necessarily a causation, between vit D levels and SARS-CoV-2 infection in adults were found in a number of studies. Ilie et al. searched the potential association between mean levels of vit D in 20 European countries and morbidity and mortality caused by COVID-19. It has been showed that the higher the vit D levels in a specific country was associated not only with lower number of cases diagnosed with COVID-19 but also with lower mortality per million population in that country (3). Additionally, vit D levels seems to be associated with a worse progression of the course of COVID-19 in the hospitalized patients (32). It still remains unclear the exact mechanism of the relationship between vitamin D and SARS-CoV-2 in younger people.

### Multisystem Inflammatory Syndrome in Children (MIS-C) and Vitamin D

The risk factors for development of MIS-C (16) correspond with those for occurrence of low vit D levels in children of African American, Afro-Caribbean descent and those related to the children's obesity (33–35).

#### Proposed Mechanism

In adults, retrospective studies demonstrated a correlation between vit D status and COVID-19 severity and mortality. Whether the vit D deficiency in COVID-19 is a cause or a result is still difficult to postulate based on the present evidence. Since vit D has been shown to have immunomodulatory activity on IL-6, and anti-IL-6 agents has already demonstrated beneficial role in the course of COVID-19, it has been speculated that administration of vit D supplementation could be beneficial for COVID-19 progression (36, 37). Up to date, no published research has addressed the relationship between vit D and MIS-C, compared with the extended number of published works regarding SARS-CoV-2 infection in adults.

The possible role of vit D in SARS-CoV-2 infection in children could be explained initially by its antiviral activities.

The antiviral properties of vit D have successfully been tested in a wide range of viral species and in different settings. Vit D potency has been investigated against influenza infection in children (38). The administration of vit D effectively reduced cytokine levels and mortality in severe critical-ill patients (30). Furthermore, vit D significantly increased rhinovirus 1B-, rhinovirus 16- and RSV-induced interferons and interferon-stimulated gene mRNA expression and protein production. It also substantially diminished rhinovirus replication and release (39). Even though a specific antiviral role of vit D against SARS-CoV2 has not been established up to now, it can possibly has an adverse effect against viral coinfections. It has been recently reported that the rate of coinfection in COVID-19 reaches 20.5%. The most common co-infections were rhinovirus/enterovirus (6.9%), RSV (5.2%), and non–SARS-CoV-2 Coronaviridae (4.3%) (40).

#### Anti-inflammatory Action of Vitamin D

**Figure 1:**
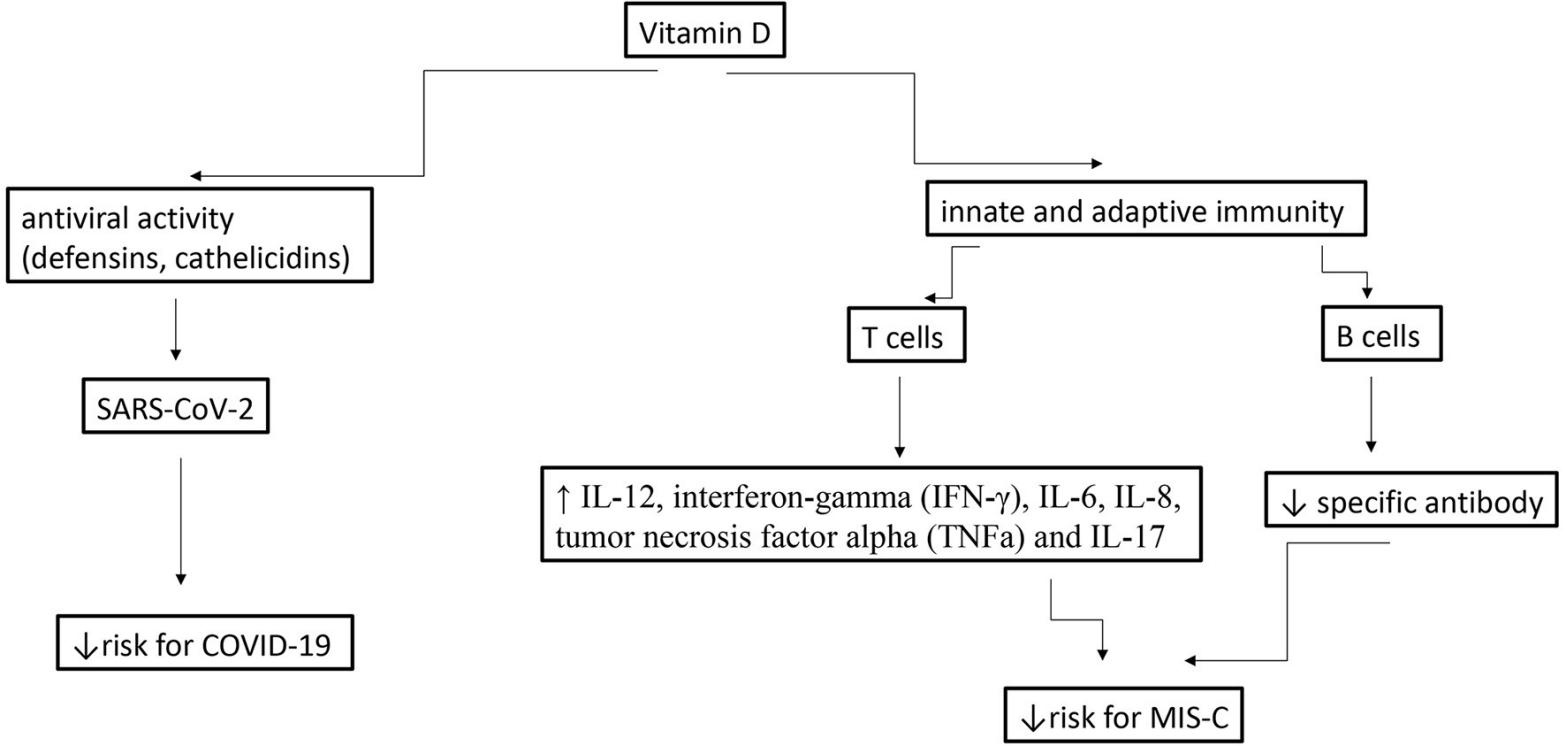
Potential effects of vitamins D during a SARS-CoV-2 infection in children and MIS-C [modified from (46)].

Since MIS-C generally occurs 3 to 4 weeks after a SARS-CoV-2 infection, consists a host-dependent reaction to a past infections (41), and this is compatible with antibody- and/or immunocomplex-mediated disease (42), we speculate that in this condition, vit D most likely plays an immunomodulatory role rather than an antiviral one. Furthermore, in the acute phase of MIS-C has been observed high levels of interleukin-1β (IL-1β), IL-6, IL-8, IL-10, IL-17, interferon-gamma (IFN-γ) and differential T and B cell subset lymphopenia (43). Vit D modulates both innate and adaptive immunity and may potentially prevents or reduces the complications associated with SARS-CoV-2 infection by increasing concentrations of anti-inflammatory cytokines (IL-10), Th2 cytokines IL-4 and IL-5, as well as by reducing concentrations of pro-inflammatory cytokines: IL-12, interferon-gamma (IFN-g), IL-6, IL-8, tumor necrosis factor alpha (TNFa) and IL-17 (44), (45). [Figure 1- modified from (46)].

Several studies shown that vit D can reduce the risk and/or the gravity of certain inflammatory reactions. Meyer et al. showed that vit D supplementation had a protective effect in association with the occurrence of Kawasaki disease in a German population (47). In a Korean retrospective review, the medical records of 91 patients with Kawasaki disease were analyzed. The patients were grouped according to vit D serum level (above and below 20ng/ml). The vit D deficient group showed statistical significant resistance to intravenous immunoglobulin (*p* = 0.023) (48). Stagi et al. assessing serum levels of 25(OH)D in 79 children with Kawasaki disease found significantly lower levels in comparison with controls (9.17 ± 4.94 vs. 23.3 ± 10.6 ng/ml, *p* < 0.0001). Vit D levels were correlated with erythrocyte-sedimentation rate (*p* < 0.0001), C-reactive protein (*p* < 0.0001), and hemoglobin level (*p* < 0.0001). Those patients who developed coronary artery abnormalities had further significantly lower vit D levels (49). Moreover, low 25(OH)D levels might contribute to the development of coronary arterial lesions (50), severity of coronary aneurysms in patients with Kawasaki disease (5) and it seems to be associated with resistance to intravenous immunoglobulin therapy (48). Activation of the STING (stimulator of interferon genes) pathway which contribute to over-immune response observed in Kawasaki disease and COVID-19, is inhibited by aspirin, intravenous immunoglobulins, vit D and anti-IL-6, potentially being beneficial in these conditions (51). Also, Boonstra et al. demonstrated that vit D3 affects Th cell polarization by inhibiting Th1 (IFN-gamma production) and augmenting Th2 cell development and consequently that results in production of their specific anti-inflammatory cytokines (52). Thus, vit D would limit the production and damaging effect of the pro-inflamatory cytokines during the MIS-C. Despite that the proof of this theory is currently insufficient, we propose measuring the vit D level at diagnosis and subsequently during the course of MIS-C in order to monitor the disease progression.

However, it is unknown as to whether vit D deficiency contributes to, or is a consequence of MIS-C. The systemic inflammation lowers circulating 25(OH)D levels (53) and this mechanism may contribute to the 25(OH)D deficiency observed in patients suffering from infectious diseases, including COVID-19 (54).

### Vitamin D as Biomarker in MIS-C

The more severe is the disease, the greater is the inflammatory process and subsequently the greater is the need for vit D (active form) with an anti-inflammatory role. Therefore, the consumption of vit D in the cells involved in immunomodulation is increased, resulting in reduced vit D level in serum (inactive form). Through this perspective, the low level of serum vit D in severe disease is a result of severity and not a predisposing factor for. Inflammatory reactions involved in the process of the diseases would reduce 25(OH)D, which would explain why low vit D levels are reported in a wide range of disorders (55, 56).

A recent study has been shown that the profiles of seven cytokines, indicated treatment responses, could be useful markers to monitor MIS-C patients undergoing treatment (18). Several of these cytokines could be decreased by vit D (45). It has yet to be shown in the clinical practice whether vit D level could be used as a biomarker for the severity of COVID-19 and MIS-C. Serial measurements during the progression of the disease could be useful in clinical practice. Additionally, the association of vit D levels with other inflammatory markers (CRP, ERS) and inflammatory cytokines (IL-6) would help clinicians to manage these.

### Vitamin D Supplementation in MIS-C

The rational of vit D supplementation in SARS-CoV-2 infection in children is based on the impact of vit D status on infections due to influenza viruses. Studying the effect of 1,200 IU/day of vit D vs. placebo on the incidence of seasonal influenza A and B among children aged 6–15 years, Urashima et al. showed reduced incidence of influenza A but not of influenza B infection (38). It seems that depending on the type of virus, the alterations at the molecular level may differ, and consequently the effect of vit D supplementation. During the 2009 pandemic of the H1N1, vit D3 supplementation did not lower the overall incidence of influenza A despite the initial benefit during the first month of treatment (57). The relationship between vit D and MIS-C and the significance of the nutritional supplementation must be proven. Vit D status has already recommended to be determined (58) and correction of low levels has been suggested (59). For this purpose, it would be useful to measure a baseline vit D level as soon as the SARS-CoV-2 infection has been detected. Then, sequential measurements, during the disease would be performed and be compared to the other inflammatory indices and to the evolution of the disease.

Increased liver enzymes during MIS-C suggests to some degree that the disturbance of liver function may reflect in the reduction of vit D 25 hydroxylation at this level. Therefore, the direct administration of the 25(OH)D3 (calcifediol) rather than vit D3 can offer a rapidly improvement of vitamin D status overcoming this impediment. Providing 25(OH)D3 to cells of immune system may contribute to reducing the severity and favorable evolution of MIS-C, particularly in children with low vit D levels. Of 50 adult patients hospitalized with COVID-19 infection and treated with of a high dose of calcifediol, one required admission to the ICU, while of 26 untreated patients, 13 required ICU admission. All patients in this study received a combination of hydroxychloroquine and azithromycin (60). The therapeutic efficacy of rapidly correcting vit D deficiency with the use of 25-hydroxyvitamin D3 [25(OH)D3] is under investigation in a clinical trial (NCT04386850) in adult patients with COVID-19. It remains to be demonstrated whether the administration of vit D supplementation is related to a potentially favorable evolution of MIS-C.

## Conclusions

In clinical practice, new biomarkers are needed for early identification of those children with MIS-C who are at increased risk of severe forms. Vit D levels could be an appropriate candidate. Additionally, correction of abnormal levels of vit D in these severe forms of MIS-C may possibly influences their evolution. 25(OH)D3 supplementation in order to raise promptly serum [25(OH)D] concentrations potentially contributes in reducing the severity of MIS-C in certain circumstance.

## Further Research

Further research is needed in this area to confirm the role vit D in MIS-C. We have to establish, through randomized controlled trials, the importance of vit D levels in prediction of MIS-C evolution. Additionally, is needed to be confirmed whether supplementation with vit D3 or 25(OH)D3 could prevent the development, progression or severity of MIS-C.

## Author Contributions

GF and VV had the conception, designed the work, and collected the data. GF, VV, IB, and EV contributed to the data analysis, its interpretation and to the article's writing and editing. LS, EV, and MZ made the critical revision of the article. All authors contributed to the article and approved the submitted version.

## Conflict of Interest

The authors declare that the research was conducted in the absence of any commercial or financial relationships that could be construed as a potential conflict of interest.
